# Dynamic myocardial CT perfusion imaging—state of the art

**DOI:** 10.1007/s00330-023-09550-y

**Published:** 2023-03-30

**Authors:** Olga Sliwicka, Ioannis Sechopoulos, Andrea Baggiano, Gianluca Pontone, Robin Nijveldt, Jesse Habets

**Affiliations:** 1 Department of Medical Imaging, Radboud University Medical Center, Geert Grooteplein Zuid 10, 6525 GA Nijmegen, The Netherlands; 2 Radboud Institute for Health Sciences, Radboud University Medical Center, Nijmegen, The Netherlands; 3 Department of Cardiovascular Imaging, Centro Cardiologico Monzino IRCCS, Milan, Italy; 4 Department of Clinical Sciences and Community Health, University of Milan, Milan, Italy; 5 Department of Biomedical, Surgical and Dental Sciences, University of Milan, Milan, Italy; 6 Department of Cardiology, Radboud University Medical Center, Nijmegen, The Netherlands; 7 Present Address: Department of Radiology and Nuclear Medicine, Haaglanden Medical Center, The Hague, The Netherlands

**Keywords:** Coronary artery disease, Dynamic myocardial computed tomography perfusion, Coronary computed tomographic angiography, Computed tomography perfusion

## Abstract

**Abstract:**

In patients with suspected coronary artery disease (CAD), dynamic myocardial computed tomography perfusion (CTP) imaging combined with coronary CT angiography (CTA) has become a comprehensive diagnostic examination technique resulting in both anatomical and quantitative functional information on myocardial blood flow, and the presence and grading of stenosis. Recently, CTP imaging has been proven to have good diagnostic accuracy for detecting myocardial ischemia, comparable to stress magnetic resonance imaging and positron emission tomography perfusion, while being superior to single photon emission computed tomography. Dynamic CTP accompanied by coronary CTA can serve as a gatekeeper for invasive workup, as it reduces unnecessary diagnostic invasive coronary angiography. Dynamic CTP also has good prognostic value for the prediction of major adverse cardiovascular events. In this article, we will provide an overview of dynamic CTP, including the basics of coronary blood flow physiology, applications and technical aspects including protocols, image acquisition and reconstruction, future perspectives, and scientific challenges.

**Key Points:**

*• Stress dynamic myocardial CT perfusion combined with coronary CTA is a comprehensive diagnostic examination technique resulting in both anatomical and quantitative functional information.*

*• Dynamic CTP imaging has good diagnostic accuracy for detecting myocardial ischemia comparable to stress MRI and PET perfusion.*

*• Dynamic CTP accompanied by coronary CTA may serve as a gatekeeper for invasive workup and can guide treatment in obstructive coronary artery disease.*

**Supplementary Information:**

The online version contains supplementary material available at 10.1007/s00330-023-09550-y.

Worldwide, coronary artery disease (CAD) results in significant cardiovascular morbidity and mortality, with it being the most common single cause of death among adults [1]. In patients with suspected CAD, coronary computed tomographic angiography (CTA) has an established diagnostic and prognostic role [2, 3] with a Class IIA level of evidence according to the latest European Society of Cardiology (ESC) guidelines [4]. However, there are some challenging settings, such as patients with severe calcifications or with previous stent implantation [5], in which coronary CTA alone could be insufficient for a definitive diagnosis. In this regard, the recent introduction of dynamic myocardial computed tomography perfusion (CTP) imaging, in combination with coronary CTA, can serve as a gatekeeper for invasive workup [6], provides added diagnostic value (sensitivity 0.86–0.96, specificity 0.74–0.84) in patients with previous coronary stents and is a cost-effective method for the detection of obstructive CAD in patients with previous stenting [7]. Moreover, CTP has incremental diagnostic accuracy *(*sensitivity 78%, specificity 73%) in patients across severity spectra of pre-test probability of CAD and coronary artery calcification. In patients with severe coronary calcification (coronary artery calcium score  ≥400), combined CTA-CTP has better diagnostic accuracy than CTA and CTP alone [8]. This simultaneous anatomical and functional assessment has comparable diagnostic accuracy to other non-invasive perfusion modalities, such as stress magnetic resonance imaging (MRI) and positron emission tomography (PET) perfusion, for diagnosing ischemia [9], providing insights on coronary plaque stenoses and myocardial blood flow [10, 11]. In this paper, we will discuss several aspects necessary to understand dynamic CTP: basics of coronary blood flow physiology, clinical applications, and technical aspects, including protocols, image acquisition, and reconstruction. This overview will conclude with discussions on future perspectives and remaining scientific challenges.

## Coronary blood flow and its measurement techniques

Coronary blood flow constitutes 4 to 5% (~225 mL/min) of the total cardiac output and is regulated entirely by intrinsic mechanisms. This intrinsic regulation, preventing ischemia, still holds even when stenosis exceeds 85–90% narrowing [10]. At rest, about 70–75% of the oxygen in the blood is extracted by the myocardium from the coronary arterial blood flow. To compensate for the minimal residual oxygen extraction capacity available under stress conditions, myocardial blood flow (MBF) increases [12]. Depending on their severity, fixed vessel narrowing and abnormal vascular tone may lead to an imbalance between myocardial demand for oxygen and its supply, initiating an ischemic cascade. Nowadays, both invasive and non-invasive methods can be used to assess the different steps in this ischemic process. Among them, different imaging modalities can be used, but the sensitivity and specificity of what they can depict, and therefore the stage at which they are useful, vary.

## Non-invasive measurement techniques

Several current non-invasive imaging modalities—PET, dual-energy CT (DECT) perfusion, single photon emission computed tomography (SPECT), cardiac magnetic resonance (CMR) imaging, and the emerging dynamic CTP—can be used to depict perfusion defects. SPECT and PET have mean sensitivity of 88.3% and 92.6% respectively to confirm  >50% stenosis of any epicardial artery in patients with known or suspected CAD compared to coronary angiography as a reference standard [13]. Furthermore, CMR also has high accuracy to detect hemodynamically significant CAD [14], provides effective risk stratification for patients with stable chest pain [15], and has been proven to be a cost-effective strategy in patients with chest pain and suspected CAD [16]. On the other hand, DECT perfusion can be performed in a static and dynamic manner. Most currently available CT systems allow only static DECT perfusion imaging. Static DECT perfusion can be performed in rest-only, stress-rest, or stress-only protocols. The sensitivity and specificity of static DECT Perfusion in literature were 75% and 95%, based on a meta-analysis [17]. An advantage of DECT perfusion is that this technique can significantly reduce beam hardening artefacts, which can negatively influence MBF measurements. The addition of coronary CTA to stress static DECT perfusion can result in improved sensitivity to 93% at the expense of a reduction of specificity to 86% [18]. However, these techniques have limitations that vary throughout institutions and countries: high costs, long acquisition times, and not negligible radiation dose for SPECT and PET [19]; low availability, high costs and long acquisition time for CMR; the inability of simultaneous acquisition of coronary artery data with PET, SPECT, or CMR. However, the latest generation of nuclear imaging (PET/SPECT) may allow for coronary CTA imaging in the same examination (hybrid imaging in a single setting) [20].

Based on the Society of Cardiac Computed Tomography (SCCT) 2020 CTP consensus document [21], CTP is indicated as an addition to coronary CTA in patients with a high likelihood of CAD, known CAD, prior coronary interventions, or significant coronary calcifications. In patients with acute chest pain, negative troponins at low to intermediate risk of CAD, coronary CTA alone is usually enough in managing such patients. However, if moderate stenosis (50–69% maximal coronary stenosis CAD-RADS 3) is detected, dynamic CTP performed in addition to coronary CTA may be used for the detection of a flow-limiting coronary lesion and results in significantly improved specificity [16, 17].

**Fig. 1:**
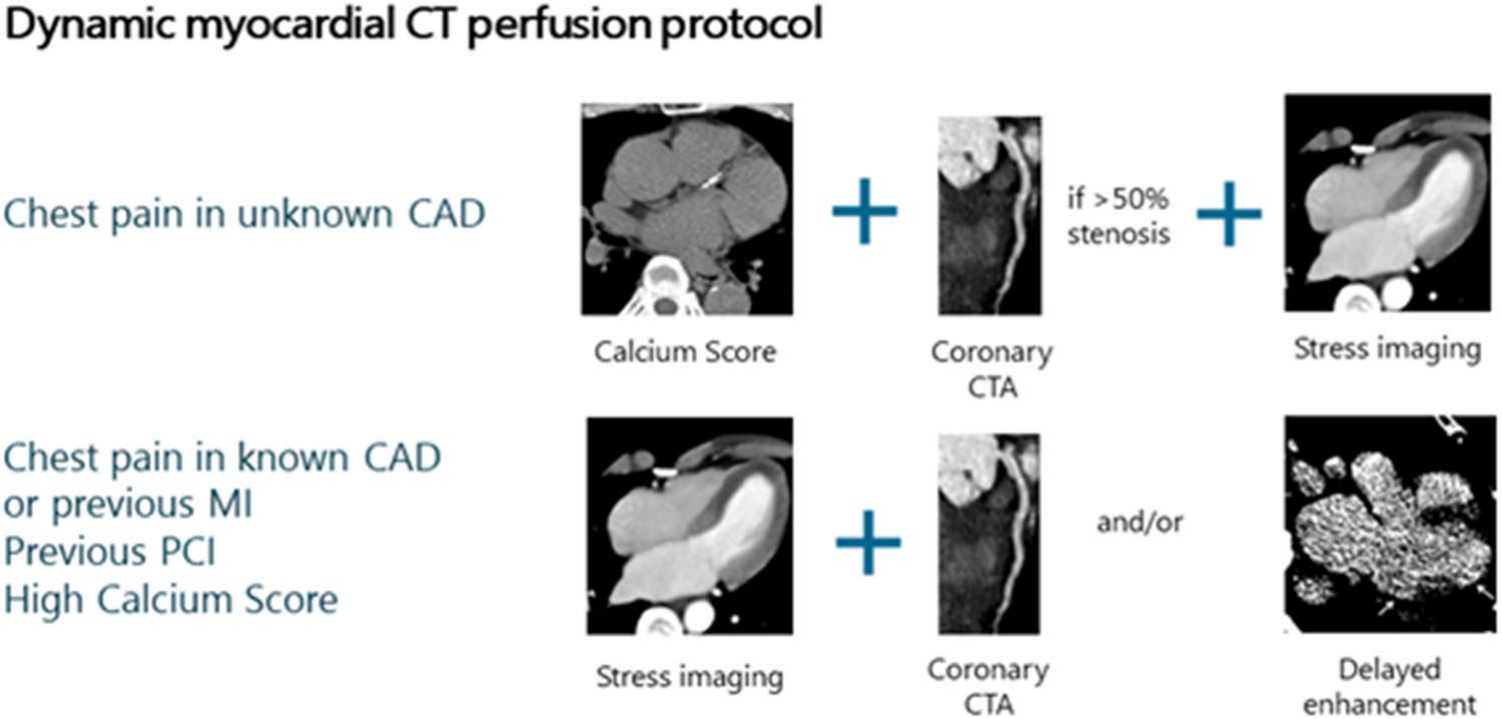
Dynamic myocardial CT perfusion protocol for two specific patient groups. CAD: coronary artery disease, CTA—CT angiography, MI—myocardial infarction, PCI: percutaneous coronary intervention

**Fig. 2:**
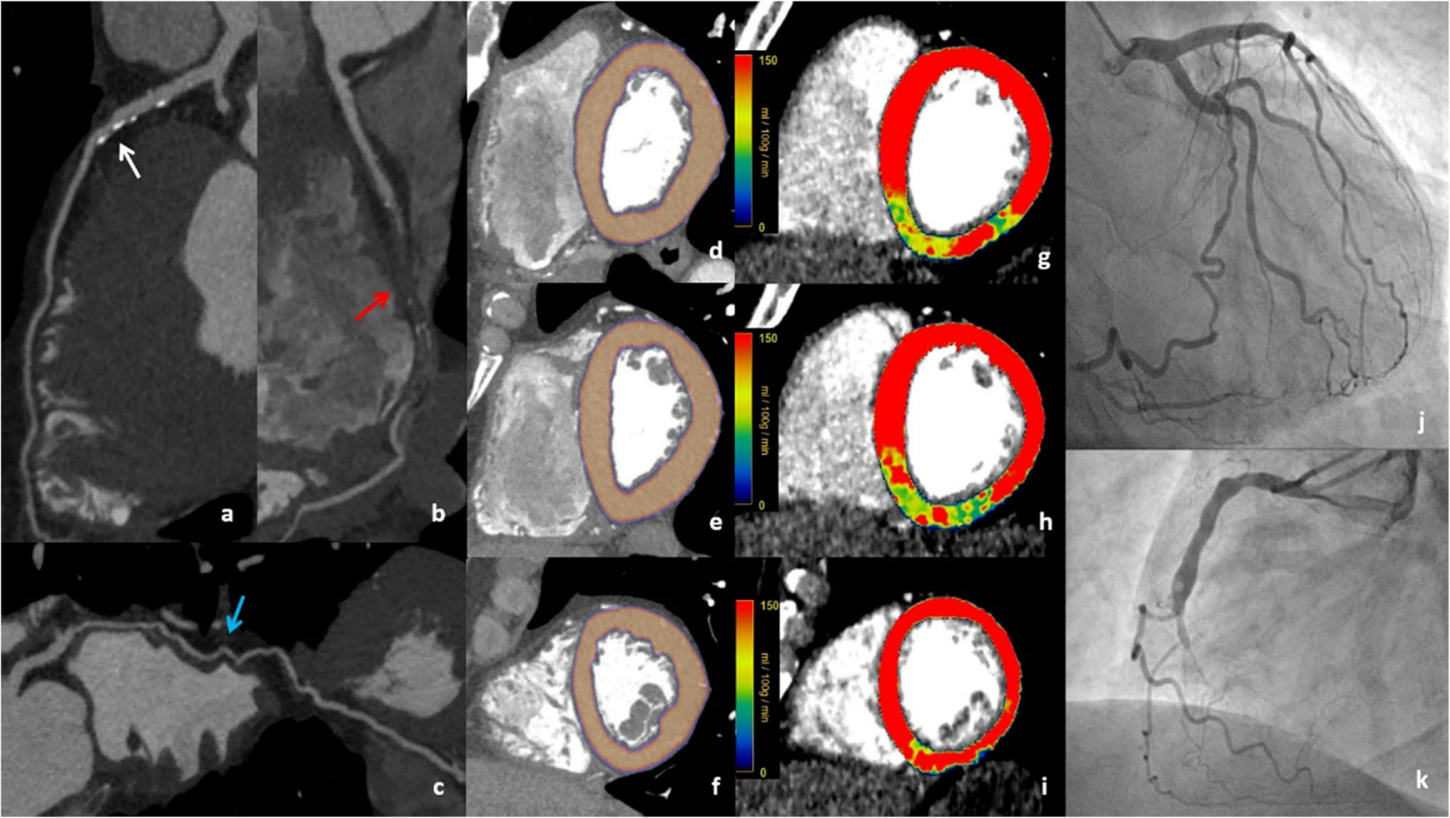
A 60-year-old man with recent onset of effort dyspnea. Coronary CTA was performed, with the evidence of moderate mid-LAD stenosis (**a**, white arrow), chronic total occlusion of mid-RCA (**b**, red arrow), and the presence of collateral supply for RCA from LCX (**c**, blue arrow). Rest myocardial perfusion assessment revealed a fully viable myocardium (**d**, **e**, **f**). After adenosine administration, a stress CTP scan performed with dynamic acquisition showed inducible ischemia at the basal to the mid inferior septum, basal to the mid-inferior wall, and basal inferolateral wall (**g**, **h**, **i**). The absence of relevant coronary stenosis at LAD, presence of collateral supply from LCX, and chronic total occlusion of mid-RCA were confirmed at invasive assessment (**j**, **k**).CTA: computed tomography angiography; LAD: left anterior descending; RCA: right coronary artery; LCX: left circumflex, CTP: computed tomography perfusion

Although in patients with previous myocardial infarction (MI) CMR is preferred due to its superior ability in infarct depiction, a CT-based exam might be an alternative (e.g. in patients with contraindications for CMR). Experience with such an approach is available (Fig. 1) but is still limited in the literature. However, conceptually CT can provide the same benefits as CMR with MBF quantification (Fig. 2) and delayed enhancement assessment, but with decreased acquisition time.

## Technical aspects of myocardial computed tomography perfusion techniques

Myocardial CTP imaging may involve two types of CT acquisitions: static and dynamic. Static CTP is snapshot imaging of the entire heart, at a single time-point of contrast enhancement, usually with the same parameters used for coronary CTA [22]. Meanwhile, dynamic CTP involves the acquisition of multiple CT datasets during contrast passage through the myocardium [11]. The greatest advantage of dynamic CTP is the absolute MBF calculation of both ischemic and healthy myocardium. Assessing the MBF allows for the evaluation of the microvascular function of the myocardium and of abnormalities in the function and structure of the coronary microcirculation that occur in many clinical conditions, including CAD and myocardial disease [23]. Furthermore, a dynamic dataset facilitates the visual assessment of perfusion deficits and simplifies clinical interpretation and distinction of the artifacts. Both techniques, discussed below, differ in hardware, acquisition time, radiation dose, costs, and patient preparation (Table 1)

**Table 1:** Comparison of static vs. dynamic myocardial CT perfusion techniques

Static CTP	Dynamic CTP
Scan duration time up to 8 heartbeats	Scan duration time up to 30 consecutive heartbeats
Moderate contrast use (70–120 mL)	
Patients’ total time in the CT lab ~15 min	Patients’ total time in the CT lab depends on acquisition protocol: - stress only or stress CTP/coronary CTA ~20–30 min, - stress CTP/rest CTP ~30–40 min including at least 15 min wait time between protocols
Shorter breath holding	Longer breath holding (~30 s)
Moderate radiation dose (5.9 mSv) [24]	Higher radiation dose (9.2 mSv*) [24]
Single dataset of acquired images	Multiple datasets of acquired images
No quantification of myocardial perfusion	Quantification of myocardial perfusion possible
Available on a 64-slice system	Requires more advanced than 64-slice system (256–320-slice) or dual-source CT

^*^- average from Table 4

## Hardware and acquisition scheme

Imaging the entire heart during dynamic CTP involves using either a wide area multidetector CT system with a stationary table or a second or third-generation dual-source CT in “shuttle mode,” with repetitive movements back-and-forth of the CT table. All major CT vendors currently have a scanner model able to perform dynamic myocardial CT perfusion.

In both static and dynamic CTP, the detection of ischemia is based on differences in attenuation in the myocardium [11]. The cut-off voxel values are relative, and various numbers have been used to describe perfusion changes in static CTP; from negative values up to 13 Hounsfield units (HU) for long-standing infarction [25], approximately 26 HU for acute ischemia [25], and  >90–100 HU for normally perfused myocardium [26–29]. However, no official cut-offs for dynamic CTP have been established. Complete acquisition of a dynamic CTP series requires a sequential performance of various different acquisitions, including stress dynamic CTP with either coronary CTA or rest dynamic CTP.

In patients with suspected CAD, first, coronary CTA followed by a stress CTP protocol is favorable: with this approach stress CTP is performed only in patients with  >50% stenosis on coronary CTA and in patients with a specific request to evaluate ischemia. Thanks to this order, the additional contrast medium and radiation could be omitted in patients without moderate (50–69%) or severe stenosis. Furthermore, in patients with a high Agatston score (>400) and patients with previous revascularization, the addition of stress dynamic CTP will improve specificity and positive predictive value compared to coronary CTA alone [30]. A similar approach (coronary CTA first and then stress Dynamic CTP) can be used for patients with acute chest pain assessed in the Emergency Department characterized by normal blood troponin levels and coronary stenoses greater than 50% of lumen diameter [31].

In patients with previous myocardial infarction or known obstructive CAD, the preferred imaging protocol includes both coronary CTA and stress dynamic CTP, while in cases with previous infarction delayed enhancement and/or rest dynamic CTP can be added.

If rest dynamic CTP is performed, the simultaneous assessment of coronary anatomy is possible with specific technical precautions (i.e. boosted coronary CTA). Rest perfusion and delayed enhancement imaging require additional radiation exposure but are of crucial help in the differentiation between ischemia and infarction [30]. Figure 1 summarizes the proposed clinical dynamic CTP algorithms.

## Medication

Vasodilator stress agents are used during stress dynamic CTP to induce hyperemia. The drug most commonly used in Europe, adenosine, is safe, relatively cheap, and effective, with a very short half-life of 2–10 s. In Asian countries, adenosine triphosphate (ATP is regularly used instead of adenosine because of its lower costs. Regadenoson is a more potent vasodilator than adenosine and exhibits selectivity for coronary circulation relative to the renal, peripheral, and mesenteric circulation. In contrast to adenosine, it is selective and does not cause negative chronotropic, dromotropic, and inotropic effects via A1 receptors [32]. Moreover, regadenoson can be used in patients with asthma and (severe) chronic obstructive pulmonary disease. The main disadvantages include its higher costs and longer half-life. However, the patients should not take caffeine 24 h before adenosine and dipyridamole infusion, as it interferes with the metabolism of these drugs. The characteristics of agents used as vasodilators in scanning protocols are shown in Table 2.

**Table 2:** Vasodilator agent used in the stress protocol

Agent	Half-life	Characteristics	Dose	Side effects	Remarks
Adenosine [10, 33, 34]	2–10 s	widely available endogenous nucleoside, nonselective activation of adenosine A_1_, A_2A_, A_2B_, and A_3_ receptors, acts on the coronary and peripheral arterioles, by modulation of sympathetic neurotransmission causes vasodilation, increases MBF, left ventricle systolic pressure, and decreases peripheral vascular resistance	140 μg/kg/min for 3 to 5 min. If the heart rate does not increase by 20 beats per minute within the first 2 min of infusion, increase in dose to 210 μg/kg	Atrioventricular block, allergy, asthma, bronchial constriction	Requires separate IV access Used mostly in Europe; Antidote: Aminophylline (usually not needed, in case of overdosing due to short half-life: stop infusion)
Regadenoson [33, 35]	33–108 min	rapid, selective A_2A_ receptor agonist, potent selective coronary vasodilator, used mostly in patients with suspected CAD, asthma, and severe chronic obstructive pulmonary disease	Intravenous bolus at a fixed dose of 400 μg/5 mL	Flushing, chest pain, dyspnea	Does not require separate IV access [11] * Used mostly in the USA; Patients require longer monitoring; Antidote: Aminophylline, may be used to accelerate the recovery of the patient
Dipyridamole [36]	40 min	non-nitrate coronary vasodilator, inhibits platelet aggregation and likely inhibits adenosine deaminase, blocks the uptake and metabolism of adenosine by erythrocytes and vascular endothelial cells. [37]	5 mg/mL	Hypotension	Requires separate IV access, coffee and tea can decrease the effectiveness

^*^ In clinical practice two intravenous canulae are required (especially using adenosine infusion), in case of need for rapid medical intervention (e.g. adenosine-induced AV block)

Other drugs, such as sublingual nitroglycerine and β-blockers are administered before coronary CTA to increase coronary CTA accuracy. Nitroglycerine [38] affects epicardial coronaries by vasodilatation and improves the visualization of stenosis, but in some cases may also decrease hypoperfusion [39, 40]. Although there are conflicting reports on the use of β-blockers resulting in the masking of ischemia or suggesting their strict contraindication in other perfusion techniques (SPECT) [22, 41–43], there is a lack of data on whether these issues apply to dynamic CTP. Administration of short half-life intravenous β-blockers (i.e. esmolol) may be an alternative to avoid the aforementioned masking [44].

## Image acquisition

The dynamic CTP image acquisition parameters needed to visualize hypo-perfused myocardium are shown in Table 3. A successful dynamic CTP acquisition starts with an unenhanced acquisition followed by contrast bolus administration to evaluate the first pass perfusion with approximately 30 consecutive heartbeats. This process is performed automatically by the current available CT systems. The acquisition is performed with prospective ECG gating during the systolic or diastolic phase of the cardiac cycle, with tube voltage correlated to the patient’s body weight. There is currently no preferred cardiac phase in dynamic CTP imaging. Most previous studies use the diastolic phase; however, the presence of large volumes of the high-density agent, in combination with the spectral nature of the x-ray beam, promotes beam-hardening artifacts [24]. Some authors have performed dynamic CTP acquisitions in systole, which is less influenced than the diastole by a high heart rate and is considered ideal to assess the presence and extension of perfusion defects thanks to the increased wall thickness. Nevertheless, it must be noted that systolic phases are more susceptible to motion artifacts, one of the main artifacts potentially affecting diagnostic accuracy. On the other hand, the presence of ischemia reduces the presence of motion in the left ventricular wall. This can facilitate the co-registration for dynamic CTP and resting coronary CTA. However, when coronary CTA is followed by stress CTP, possible cross-contamination of contrast may occur. The contrast remaining in the scar area of the myocardium may result in false positive findings in the following stress imaging [11].

**Table 3:** Example of image acquisition and post-processing parameters used in dynamic CT perfusion protocols in Radboudumc (Aquillion One PRISM Scanner, Canon), Centro Cardiologico Monzino (Revolution Scanner, GE Healthcare), UMC Groningen (SOMATOM Force Dual Source, Siemens Healthcare) and an example protocol from Philips (iCT Philips, Philips Healthcare)

Vendor	Canon	GE Healthcare	Siemens	Phillips
Scanner type	Aquillion One PRISM	Revolution CT	SOMATOM force dual source (sequential shuttle mode)	iCT
Parameter				
Tube voltage (kV)	For BMI <30: 80 For BMI 30–35: 100	100	70–80	80
Tube current (mA)	100	150	Patient dependent	mAs dependent
Quality reference mAs	n/a	n/a	440	n/a
Effective tube current-exposure time product (mAs)	Patient dependent	Patient dependent	Patient dependent	Patient dependent
Computed tomography dose index volume (mGy)	For 100 kV: 24, For 80 kv: 10	3 phases*: 1^st^ phase: 9.73; 2^nd^ phase: 10.79; 3^rd^ phase: 6.48	Patient dependent	6.3 mGy/100 mAs
Rotation time (s)	0.275	0.28	0.25	0.27
Nominal focal spot size (mm)	0.9 × 0.8	1.0 × 0.7	0.8 × 1.1	1.1 × 1.2
Z-axis heart coverage (mm)	160	160	116	80
Scan mode, collimation (mm)	120 × 0.5 – 160 × 0.5	Smart collimation	2 × 96 × 0.6	64 × 1.25
Default in-plane reconstruction field of view (mm)**	220	200	heart size dependent	190
Reconstruction method	AiCE	ASIR-V	ADMIRE	iDose4
Reconstruction kernel	Cardiac	Standard	Qr36	CC
Reconstruction matrix	512 × 512	512 × 512	512 × 512	512 × 512
Slice thickness, increment (mm)	0.5/0.5	1.25/1.25	3.0/1.0	2 to 3/2 to 3
Number of detector rows	320	256	*384* (2 × 192)	128 (256)
Example of post-processing software	Vitrea Dynamic Myocardial Perfusion	GE CardiQ Xpress Dynamic Perfusion	Syngo CT Myocardial Perfusion	Dynamic Myocardial Perfusion software in IntelliSpace Portal
Default myocardial window width and level (WW/WL)***	150/300	800/200	200/600	750/90

*n/a* not applicable, *AiCE* Advanced intelligent Clear-IQ Engine, deep learning-based reconstruction algorithm, *ASIR-*V iterative hybrid model-based reconstruction algorithm., *ADMIRE* Advanced Modelled Iterative Reconstruction, *iDose4* 4th generation iterative reconstruction technique. * Dynamic myocardial CT perfusion protocol in GE Revolution CT has three phases of scanning; these CTDIvol values represent average values during the mentioned phase of scanning. **field of view may be adjusted, ***default window settings used by the hospitals

The number of acquired images depends mainly on the patient’s heart rate, with image acquisition performed at every (1RR), second (2RR), third (3RR), or fourth (4RR) heartbeat. Emerging literature suggests that the use of the 2RR scheme results in non-inferior image quality while halving the dose [36, 45]. However, the use of temporal under-sampling may lead to the underestimation of the true MBF and may therefore introduce errors in its quantification in terms of maximum enhancement, maximum slope, or hybrid deconvolution [24, 45].

## Image reconstruction

Before reconstruction, the most common phenomena needing correction are image artifacts, including motion artifacts, due to high heart rate [11], respiration, spatial misalignment, and beam-hardening artifacts, with the latter may mimicking perfusion defects [10]. For these reasons, datasets usually undergo dedicated post-processing algorithms to improve accuracy and image quality. With a whole-heart scanner, a motion compensation non-rigid image registration algorithm is applied to remove motion artifacts due to the free breathing of patients during the acquisition [46] with a dual source scanner using the “shuttle-mode” technique, a spatial-diffusion filter reduces spatial misalignment and motion artifacts.

Reconstruction of dynamic CTP images into 1-mm slices with 1-mm intervals [47] is performed using advanced non-linear, partial, or full, model-based iterative reconstruction algorithms, in addition to artificial intelligence [48] and temporal averaging techniques. The use of artificial intelligence-based reconstruction has also been shown to allow for an acquired slice thickness of 0.5 mm while resulting in reduced artifacts and an improved signal-to-noise ratio [49]. For image evaluation, imaging datasets with a slice thickness of 5–8 mm are reconstructed as recommended by the SCCT Expert Consensus document [21]. Moreover, noise filtering algorithms may be used for reconstruction with noise reduction filters. One of them, the 4D similarity filter [50], applied to dynamic CTP images, provides noise reduction by averaging voxels corresponding to similar tissue types. Its application results in a more natural texture depiction with sharp vessel contours compared to the one obtained with conventional local spatial filtering [50].

Dynamic CTP post-processing takes from 15 to 30 min and is performed semi-automatically on a dedicated workstation with the dynamic myocardial perfusion application (Appendix 1). Some vendors require selecting a target phase with optimal contrast enhancement in the left and right ventricles before segmentation, while cardiac axis and wall contours are extracted automatically. Manual adjustments, if needed, may be performed by choosing the correct axis, alignment, and contouring of the ventricle and pointing out the highest value on the contrast inflow time density curve before computing the results. The reconstructed images are displayed in a short axis (apical, midventricular, basal slices) and 2-, 3-, and 4-chamber long-axis views. Window width and window level may be adjusted afterward, depending on the enhancement and noise. Furthermore, the SCCT Expert Consensus document advises using a narrow window width of 200–300 and a level setting of 100–150 [21]. Finally, every image plane can be reconstructed into a 4D dataset, providing a significant advantage for dynamic CTP over other non-invasive modalities such as CMR.

## Radiation dose

The relationship between radiation dose, scanning protocol, and diagnostic accuracy for different CTP studies is shown in Table 4. Although the later generation scanners are preferred to reduce the radiation dose, the radiation exposure in dynamic CTP varies among protocols and vendors, having a mean effective dose of 9.2 mSv (range of 4.6–12.8 mSv). Currently, limiting the temporal sampling rate is a promising option to decrease the radiation dose but, as mentioned, may underestimate true MBF, thus decreasing diagnostic accuracy [24, 45] and therefore requires further research before definitive clinical implementation. However, recent reports on motion-immune perfusion imaging [51] suggest that this technique may improve quantitative accuracy while also reducing the radiation dose. The motion immune technique defines the entire myocardium as a single large volume-of-interest where measurements are made before hyperemic transit, solving the problem of perfusion underestimation. Hence, this solution provides the diagnostic advantages of dynamic CT perfusion while mathematically eliminating the negative impacts of motion and registration on MBF quantification accuracy [51].

**Table 4:** Overview of dynamic CTP studies. The order indicates studies with most to least study participants

Author	Year	Ref. No	No. of patients	CT technology	Comparator	Analysis	BMI (kg m^−2^)	Effective dose (mSv)	Sens. %	Spec. %	PPV %	NPV %	Stress agent
Rest/stress protocol													
Magalhaes et al	2015	[8]	381	320-row dual-source CT	ICA + SPECT	Visual assessment Qualitative metrics	27 (24–30)	5.31 (3.81–6.04)	58	86	87	55	Adenosine 0.14 mg/kg/min
Lubbers et al	2018	[52]	268	Second- and third-generation dual-source CT	Coronary CTA	MBF quantification Visual assessment	28 ± 5	10.6 ± 6.3	n/a	n/a	n/a	n/a	Adenosine 0.14 mg/kg/min
Meinel et al	2017	[53]	242	Second-generation dual-source CT	Coronary CTA	Qualitative interpretation of MBF and MBV Visual assessment	n/a	n/a	n/a	n/a	n/a	n/a	Adenosine 0.14 mg/kg/min
Vliegenthart et al	2016	[54]	98	Second-generation dual-source CT,	Coronary CTA	Qualitative and quantitative analysis of MBF and MBV	n/a	9.4 to 11.1	n/a	n/a	n/a	n/a	Adenosine 0.14 mg/kg/min
Pontone et al	2019	[87]	85	third-generation CT scanner	ICA + FFR	MBF quantification	26.7 ± 4.8	2.8 ± 1.2 (rest protocol) 5.3 ± 0.7 (stress protocol)	73	86	72	87	Adenosine 0.14 mg/kg/min (over a 4 min period)
Rossi et al	2014	[56]	80	Second-generation dual-source CT scanner	CTCA and semi-automatic quantitative CT	Visual comparison MBF index interpretation	27 ± 4	9.4	88	90	77	95	Adenosine 0.14 mg/kg/min
Wichmann et al	2016	[57]	71	Second-generation dual-source CT,	n/a	MBF and MBV quantification Visual assessment	n/a	n/a	100–81.3	100–90.9	100–72.2	100–94.3	Adenosine 0.14 mg/kg/min
Baxa et al	2015	[58]	54	Second-generation dual-source CT,	Coronary CTA + ICA	Visual assessment Color-coded perfusion maps (MBF and MBV)	25.7 ± 6	8.9 ± 2.4	97	95	95	98	Regadenoson 0.4 mg
Kono et al	2014	[59]	42	128-slice dual-source CT	ICA + FFR (cut-off < 0.8)	MBF quantification (MBF ratio)	26.2 ± 2.6	9.4	97.8	69.6	75.9	9	Adenosine 0.14 mg/kg/min
Ho et al	2015	[60]	35	128-slice dual-source CT	n/a	MBF quantification	23.91 ± 3.49	12.91 ± 4.32	n/a	n/a	n/a	n/a	Dipyridamole 0.56 mg/kg/min (over a 4-min period)
Bamberg et al	2014	[61]	31	Dual-source CT	ICA + FFR	MBF and MBV quantification (model-based parametric deconvolution technique)	26.56 ± 4.12	11.08	100	75	92	100	Adenosine 0.14 mg/kg/min
Wang et al	2012	[62]	30	128-slice dual-source CT	SPECT	MBF quantification	24.3 ± 2.9	9.5 ± 1.3	100	68.8	73.7	54.1	Adenosine 0.14 mg/kg/min
Bindschadler et al	2018	[63]	27	Revolution CT-scanner	n/a	MBF quantification	31 ± 5	8 ± 1	n/a	n/a	n/a	n/a	Regadenoson 0.4 mg
Giordano et al	2017	[64]	22	Dual-source CT	Coronary CTA	MBF and HPV quantification	n/a	n/a	83	77	79	84	Adenosine 0.14 mg/kg/min
Stress/rest protocol													
Nakamura et al	2018	[65]	332	Third- and second-generation dual source CT	Coronary CTA	MBF quantification	n/a, 112 patients >25	7.1	74	72	15	98	ATP 0.16 mg/kg/min for > 3 min
Nous et al	2021	[66]	114	Third-generation dual-source CT	ICA + FFR (cut-off < 0.8)	Qualitative and quantitative analysis of MBF (per vessel)	26 ± 4	-	84	89	73	94	Adenosine 0.14 mg/kg/min
Tanabe et al	2016	[67]	149	256-slice multi-detector CT	SPECT / MRI	Qualitative and quantitative analysis of MBF	24.3 ± 3.3	10.5	n/a	n/a	n/a	n/a	ATP 0.16 mg/kg/min
Kim et al	2014	[68]	92	128-slice dual-source CT	n/a	Signal-to-noise ratio, contrast-to-noise ratio	22.3 ± 1.7	4.6–10.0 ± 1.0–1.6	n/a	n/a	n/a	n/a	Adenosine 0.14 mg/kg/min
Yi et al	2018	[69]	56	Third-generation dual-source CT	Coronary CTA	Quantitative and qualitative analysis of MBF	25.6	5.98 ± 2.01	94.9	77	95.1	91.6	ATP 0.14 mg/kg/min
Goto Y et al	2017	[70]	51	Dual-source CT,	ICA + FFR (cut-off < 0.8)	MBF quantification	23.5 ± 3.6	11.3 ± 2.3	81.1	60	64.7	78.9	Adenosine 0.14 mg/kg/min
Nishiyama et al	2019	[6]	38	256-slice multidetector CT	ICA + FFR (cut-off <0. 75)	MBF quantification	24.0 ± 3.5	10.2 ± 1.2	83	93	77	95	ATP 0.16 mg/kg/min
Alessio et al	2019	[71]	34	256-slice single-slice CT	82-Rubidium PET	MBF quantification	31 ± 5	8.4 ± 1.1	75	83	n/a	n/a	Regadenoson 0.4 mg
Obara et al	2018	[72]	27	320-row multidetector CT	Coronary CTA + ICA	MBF and CFR quantification Visual assessment	24 ± 3	11.8 ± 2.4	88	82	88	82	ATP 0.16 mg/kg/min
Kikuchi et al	2014	[73]	7	320-row multidetector CT	PET	MBF quantification Visual assessment	26.8 ± 2.6	12.8 ± 2.9	87.5	92.3	n/a	n/a	ATP 0.16 mg/kg/min

*n/a* not applicable, *MBF* mean blood flow, *CFR* coronary flow reserve, *HPV* hypoperfused volume, *MBV* myocardial blood volume, *MRI* magnetic resonance imaging, *ATP* adenosine triphosphat

Moreover, noise-reducing filters, such as four-dimensional similarity filtering (4D-SF), may be another option to decrease radiation. 4D-SF after deep-learning-based reconstruction improves image quality, lowering the image noise and artifacts while improving cardiac contour sharpness and diagnostic ability, possibly enabling dose reduction in dynamic CTP imaging in patients with suspected chronic coronary syndrome [49].

## Image analysis

To analyze and interpret dynamic CTP images, the visual assessment of perfusion defects, primarily used in clinical practice, can be supported by advanced algorithms calculating the MBF. In patients with 4D reconstructed datasets, the perfusion deficits are compared across stress and rest. Scar remnants of previous (irreversible) infarction do not enhance in either the stress or rest datasets. On the other hand, perfusion deficits visible during stress but invisible at rest are (reversible) ischemic, with their size correlating with hemodynamic relevance and severity of luminal stenosis [22].

**Fig. 3:**
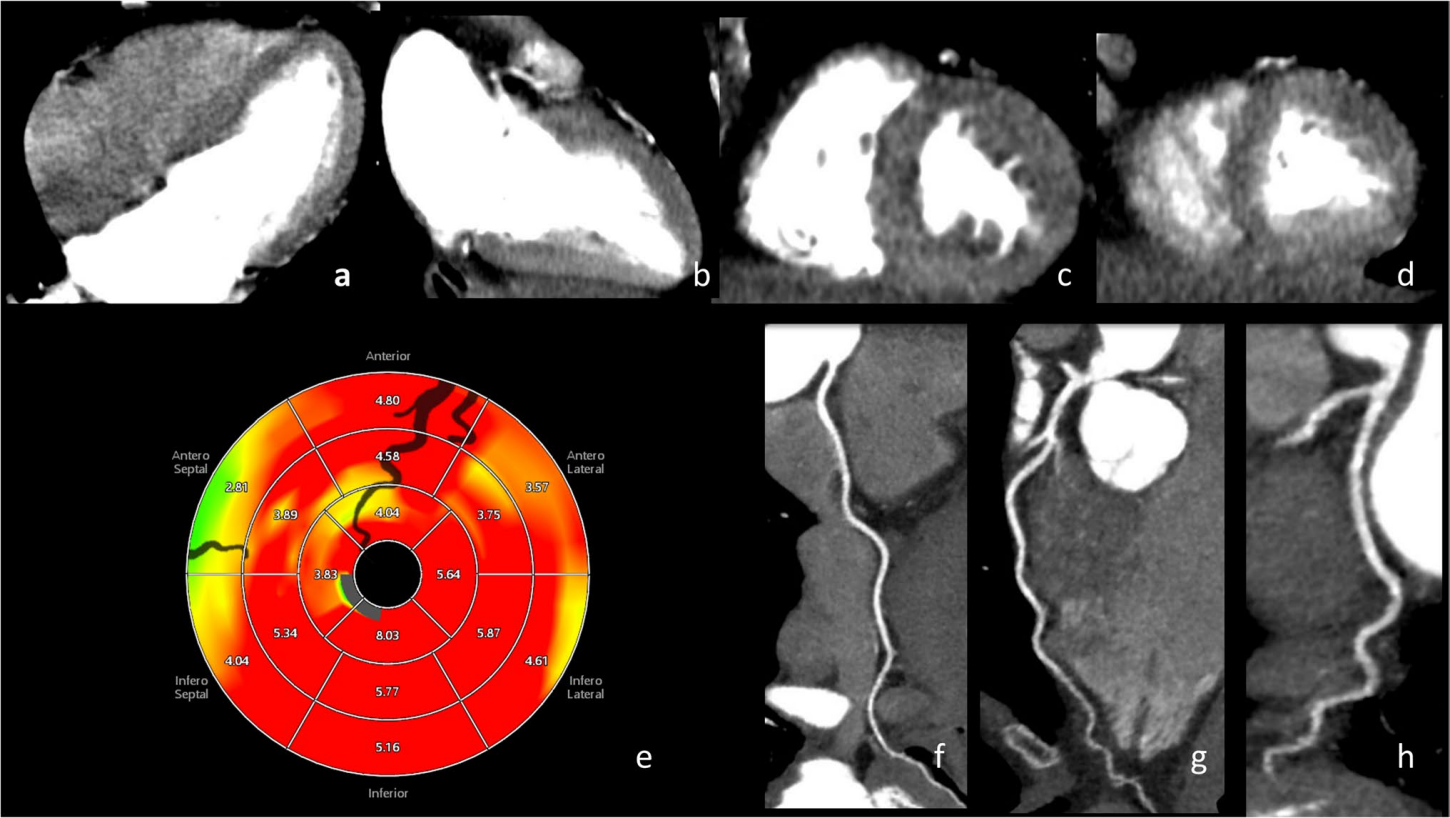
A 59-year-old female ex-smoker with familial hypercholesterolemia and relevant family history of cardiovascular disease presented to the hospital with atypical angina pectoris for the first time. She was referred for stress dynamic myocardial CT perfusion with FFR-CT. The coronary CTA showed no calcifications in the coronary arteries (RCA – **f**, LAD – **g**, LCX – **h**) or valves, except small calcifications in the aortic root and ascending aorta. In dynamic CTP: a normal stress perfusion CT images. Stress perfusion with adenosine (140 μg/kg) showed no evidence of obstructive epicardial coronary artery disease (**a**, **b**, **c**, **d**, **e**): no anomalies at the origin of the coronary arteries. No stenosis or plaques in the RCA, LAD, or LCX. CT-FFR with no significant pressure difference across arteries. In the CT extracardiac findings, the patient had a small thoracic hernia most probably responsible for the atypical chest pain. Upper row: dynamic myocardial CTP imaging: **a** four-chamber view, **b** two-chamber view; short axis: **c** basal view, **d** midventricular view. Lower row: **e** dynamic CT perfusion map with artery visual representation (black shadows), **f** coronary CTA RCA, **g** coronary CTA LAD, **h** coronary CTA LCX

**Fig. 4:**
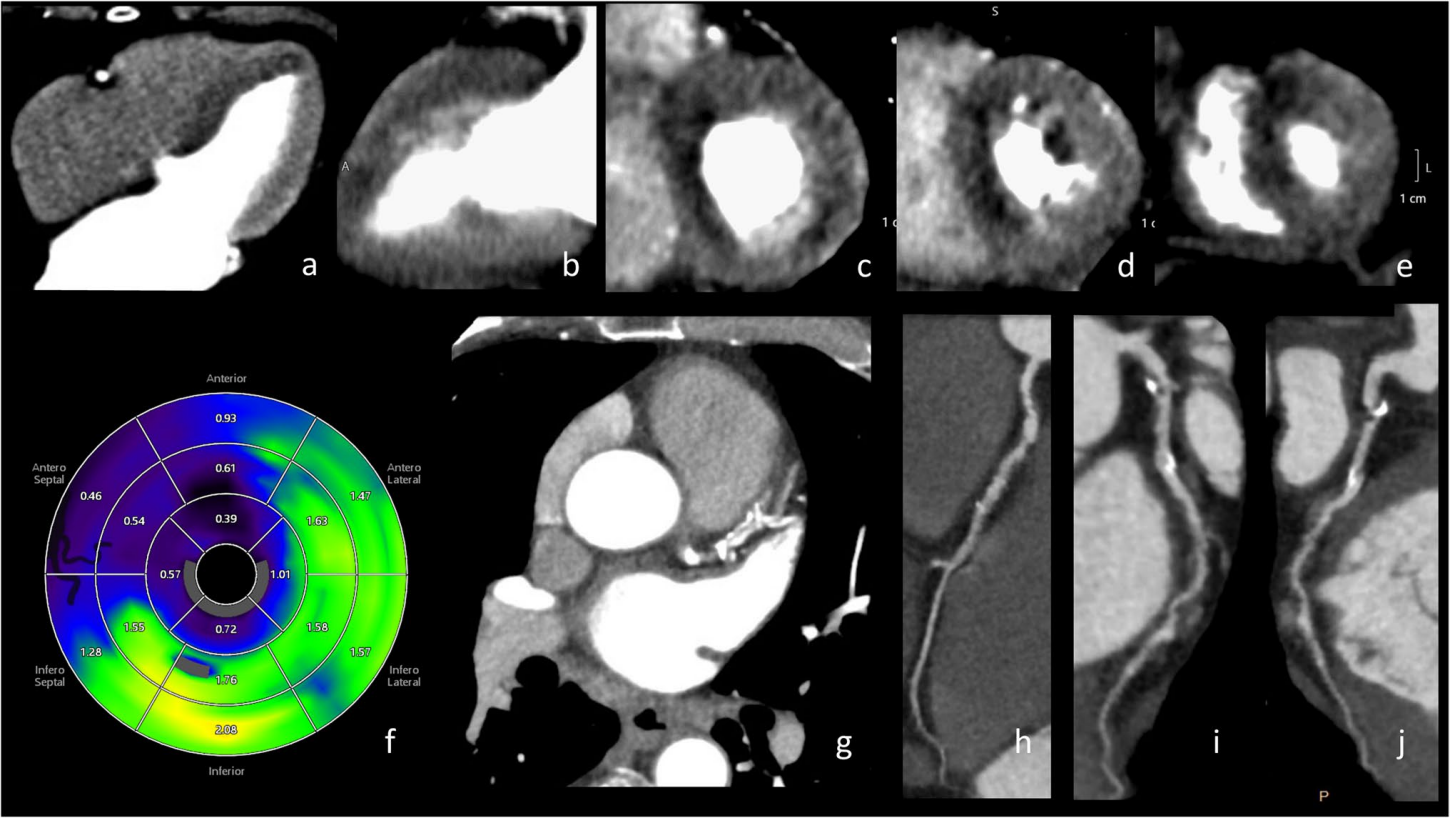
A 70-year-old male with poorly-regulated hypertension presented to the outpatient clinic with stable angina pectoris. Three months prior, he experienced chest pain, but acute coronary syndrome was excluded at the ER. The patient underwent a CT examination with calcium score, coronary CTA, and dynamic stress CTP protocol. The calcium score was 278.83 (62^nd^ percentile). Coronary CT angiography showed 50% stenosis in the distal left main artery (**i**), stenosis of 70–99% in the proximal (**i**), the mid-LAD (**i**), and origo LCX (**j**). In the proximal RCA (**h**), the intermediate stenosis based on the non-calcified plaque was 50%-69%. In the mid-LAD and mid-LCX: plaques with high-risk plaque features (positive remodeling and hypo-attenuated plaques). Positive adenosine stress CT perfusion with extensive ischemia in LAD territory in multiple segments (**a**–**f**). Furthermore, the ischemia in the basal slice in antero/inferolateral segments could be related to the left main stenosis  >50% and significant stenosis in proximal LCX (**c**). Furthermore, diffuse low-stress MBF values in all segments, LAD territory most severely affected, supporting the diagnosis of multiple vessel disease. The patient underwent invasive angiography that confirmed significant multiple vessel disease in LAD (proximal and mid-segment,  >70% stenosis) and proximal LCX (>70%). The intermediate lesion in the RCA was FFR-negative (FFR 0.83). The stenosis in the distal left main was 50%. The patient was referred for CABG. Upper row: dynamic myocardial CTP imaging: **a** four-chamber view, **b** two-chamber view; short axis: **c** basal view, **d** midventricular view, **e** apical view. Lower row: **f** dynamic CT perfusion map, **g** coronary CTA slice with LAD stenotic changes, **h** coronary CTA RCA, **i** coronary CTA LAD, and **j** coronary CTA LCX

Quantification of MBF provides additional diagnostic information thanks to the evaluation of blood inflow to the myocardium based on the time attenuation curve (TAC) for the region of interest [22]. In the literature [30], several MBF quantification approaches have been described. There were a compartment model, a maximal upslope model, a deconvolution model [74], upslope analyses (semi-quantitative) [26, 75], a Myocardial Segmental Perfusion Index [75], a 3D segmental Volumetric Perfusion Index [75] and finally, a 17-segment model [76] among them. In the last one, MBF results are displayed as color-coded perfusion maps, divided into 17 myocardial segments corresponding to the vessel bed of the left anterior descending artery (LAD), right coronary artery (RCA), and left circumflex artery (LCX). Stress MBF perfusion is usually displayed in red above 4.0 mL/g/min, while cut-off values for ischemia, typically in blue, vary in literature but generally are below 1.0 mL/g/min (Figs. 3 and 4). Furthermore, MBF calculation may also be adjusted in patients with a high MBF value by the Renkin-Crone method [73] because this method can convert MBF from CT to true MBF [77].

In previous studies (Table 4), absolute MBF measurements derived from dynamic CTP have been shown to outperform other quantitative parameters, like myocardial blood flow ratio and myocardial blood volume [78], for the detection of significant CAD [6, 72, 79, 80]. In recent reports, however, the stress myocardial blood flow ratio has been introduced and shown to be an accurate method to identify flow-limiting lesions [27, 81], and to increase specificity for detection of significant CAD to 91%, when compared with invasive FFR [27]. Both absolute MBF and MBF-ratio have excellent diagnostic performance (with reference to FFR) and outperform visual analysis for the detection of myocardial ischemia [82].

## Diagnostic accuracy

An overview of the diagnostic accuracy of dynamic CTP is shown in Table 4. A recent report shows that static CT perfusion has low sensitivity with invasive FFR (≤ 0.80) as a golden standard [83]. However, a meta-analysis from 2016 by Sørgaard et al [84] suggested that static CTP has high sensitivity (85%) for detecting myocardial ischemia, especially when combined with coronary CTA. The diagnostic accuracy of dynamic CTP is comparable to that of CMR and PET, with a sensitivity of 93% (82–98%, 95% CI interval) and a specificity of 82% (70–91%, 95% CI interval) on a patient level [85] and is superior to that of SPECT [85]. Moreover, the addition of dynamic CTP to coronary CTA significantly improves specificity, raising it to 86% (76–93%, 95% CI interval) [80], improves the risk stratification of patients with stenosis [65, 86], and reduces the use of unnecessary diagnostic invasive coronary angiography (ICA) [31].

Adding fractional flow reserve CT (CT-FFR) on top of coronary CTA increases diagnostic accuracy for the detection of functionally obstructive CAD [87] and is as accurate as myocardial CTP in providing functional quantification of fractional flow reserve [55, 88, 89]. CT-FFR can be estimated based on dedicated coronary CTA, providing a functional evaluation of coronary stenosis and improving the identification of significant CAD [80, 90]. Therefore, the sequential integration of coronary CTA, CT-FFR, and dynamic CTP increases sensitivity and specificity for the detection of significant CAD [90, 91]. Furthermore, dynamic CTP alone has the highest prognostic value for major adverse cardiovascular events (MACE) (compared to coronary CTA and CT-FFR individually or a combination of the three), independent of clinical risk factors [51]. Moreover, patients with at least one perfusion defect at dynamic myocardial CTP were at increased risk of MACE (hazard ratio: 2.50; [1.34–4.65, 95% CI]; *p* = 0.004), regardless of the adjustment for clinical risk factors and coronary CTA findings [53].

## Limitations

Dynamic CTP is an emerging imaging technique that is still undergoing development and optimization. Nowadays, only a few medical centers worldwide use it clinically, as it is not routinely reimbursed and can be performed only on high-end CT equipment. Standardized validated MBF cut-off values are lacking. MBF values are strongly dependent on the algorithm used to calculate them. In clinical practice, the combination of the visual presence of a segmental perfusion deficit and MBF value  < 1.0 ml/g/min can be used to diagnose ischemia. Regarding high image quality and radiation exposure concerns, dynamic CTP imaging may be restricted to a certain maximum body mass. The maximum advised allowed body mass varies in the literature, with limiting values specified as maximum BMI (under 30 kg/m^2^ [72], 35 kg/m^2^ [78], or 40 kg/m^2^ [8, 92]), and maximum body mass (under 120 kg [92]).

Besides body mass, the presence of an implantable cardioverter-defibrillator and pacemaker leads are contraindications to undergo clinical CTP scanning because of severe beam hardening artifacts that hamper the diagnostic visual assessment of CTP images and significantly influence MBF quantification. The recent introduction of reconstruction of dynamic CTP datasets with advanced metal artifact reduction algorithms can improve image quality significantly, allowing for accurate diagnostic assessment. However, large studies evaluating the diagnostic accuracy of these advanced metal artifact reduction algorithms and the wide availability of these metal artifact reduction reconstruction algorithms are lacking.

## Staff, image examples, and reporting with structured standardized report

A cardiovascular imaging specialist is a qualified physician to perform dynamic CTP imaging after specific CTP training. Evaluation of patients eligible to undergo dynamic CTP should include clinical history, physical evaluation, and preparation for the examination. The patient should be monitored during and after the scan and during drug and contrast administration. Reporting after the study should start with coronary anatomy evaluation for coronary plaques and stenosis, including calcium score, cardiac perfusion analysis, and evaluation of extracardiac structures. Finally, the detected perfusion abnormalities and infarcted segments should be correlated to corresponding coronary CTA findings.

## Future perspectives and scientific challenges

The role of dynamic CTP is determined now for stable angina pectoris with intermediate risk and acute chest pain with negative troponins based on current scientific evidence, including meta-analysis and diagnostic trials. According to the ESC guidelines [93], invasive coronary angiography (ICA) remains the preferred technique for diagnosing non-ST elevation infarctions. Furthermore, evidence of the potential role of dynamic CTP with coronary CTA in the identification of obstructive CAD and its role in guidance for ICA is lacking. However, dynamic CTP demonstrates great potential to evaluate microvascular functions other than ischemia [94]. The CTP-MBF derived from porcine in-vivo hearts could quantify the microvascular impairment in different myocardial regions after MI and track its recovery over time (with MRI and histopathological findings as reference standards). This will facilitate a rapid approach for pathophysiological insights following MI [95]. More prospective research is needed to confirm the dynamic CTP role in microvascular disease.

Since dynamic CTP is associated with considerable radiation dose, further research is needed to decrease the dose required while maintaining image quality. This could be achieved by further optimization of the acquisition technique (e.g. by using skipped-beat acquisitions) and developing novel reconstruction or acquisition algorithms. Currently, the ASTRA4D algorithm shows promising clinical results in noise reduction and motion elimination in low-dose 4D CTP by combining local temporal regression and deformable image registration and improving spatiotemporal ischemia differentiation [96]. Moreover, accelerating dynamic CTP post-processing using artificial intelligence tools could improve clinical applicability.

## Conclusion

Dynamic CTP combined with coronary CTA has emerged as a comprehensive non-invasive diagnostic technique resulting in both anatomical and quantitative functional information, providing insights not only on coronary plaque morphology and stenosis but also on myocardial blood flow. This anatomical and functional CT-based approach warrants a diagnostic accuracy comparable to CMR and PET, and superior to SPECT, for detecting hemodynamically significant stenosis., and, in light of such favorable diagnostic performance, a growing role in the clinical management of patients with CAD is expected in the next future.

## Supplementary Information

Below is the link to the electronic supplementary material.

Supplementary file1 (PDF 166 KB)

## Abbreviations

ATP: Adenosine triphosphate
BMI: Body mass index
CAD: Coronary artery disease
CAD-RADS: Coronary Artery Disease-Reporting and Data System
CMR: Cardiac magnetic resonance
CTA: Computed tomographic angiography
CT-FFR: Computed tomography fractional flow reserve
CTP: Computed tomography perfusion
DECT: Dual-energy CT
ESC: European Society of Cardiology
FFR: Fractional flow reserve
HU: Hounsfield units
LAD: Left anterior descending artery
LCX: Left circumflex artery
LGE: Late gadolinium enhancement
LV: Left ventricle
MACE: Major adverse cardiovascular event
MI: Myocardial infarction
MRI: Magnetic resonance imaging
PET: Positron emission tomography
RCA: Right coronary artery
SPECT: Single photon emission computed tomography

## Declarations

### Guarantor

The scientific guarantor of this publication is Olga Sliwicka.

### Statistics and biometry

No complex statistical methods were necessary for this paper.

### Methodology

• Not applicable

• Not applicable

• Performed at one institution
