# Supplementary figures and images for: Dynamic myocardial CT perfusion imaging—state of the art
Source: Eur Radiol. 2023 Mar 30;33(8):5509–25. doi: 10.1007/s00330-023-09550-y (PMC10326111; doi:10.1007/s00330-023-09550-y)

## Appendix 1.

### Step-by-step performance of dynamic CT perfusion imaging.

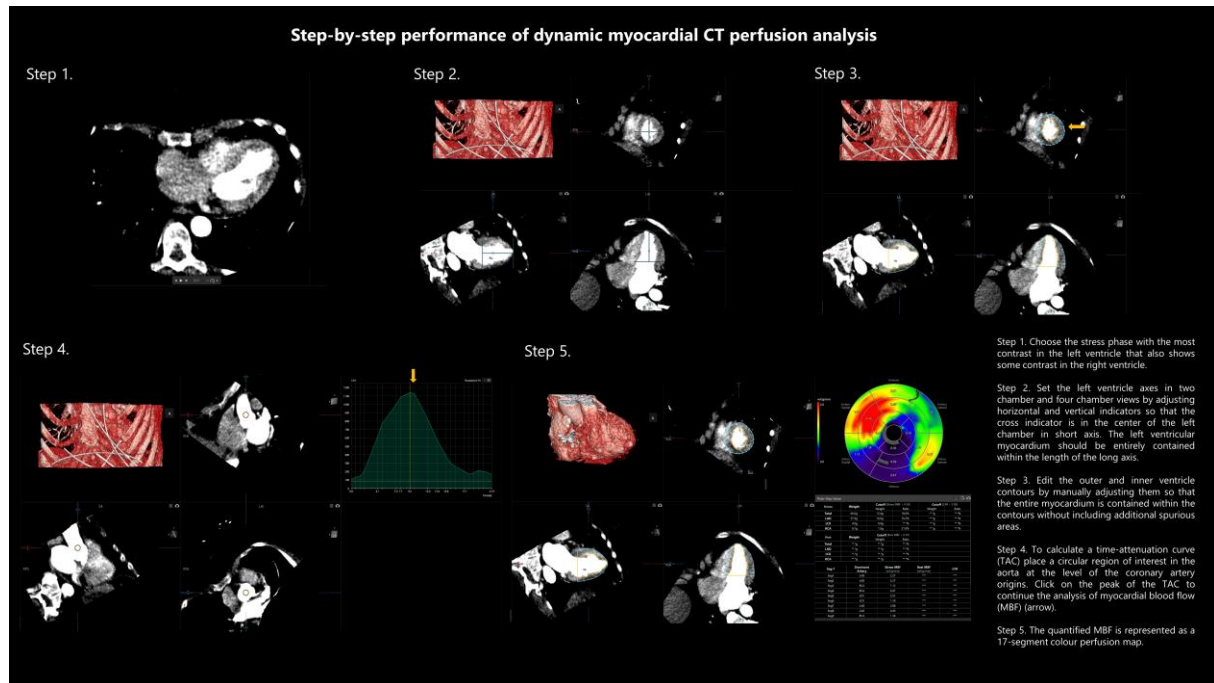

Supplement: Supplementary file 1 — Supplementary file1 (PDF 166 KB) [file 330_2023_9550_MOESM1_ESM.pdf]
